# Diffuse Alveolar Haemorrhage as a Rare Pulmonary Manifestation of Antisynthetase Syndrome: A Case Series

**DOI:** 10.3390/jcm15072555

**Published:** 2026-03-27

**Authors:** Katarzyna Królak-Nowak, Mikołaj Janiak, Tymon Putyński, Aleksandra Opinc-Rosiak, Joanna Samanta Makowska, Adam Antczak

**Affiliations:** 1 Department of General and Oncological Pulmonology, Medical University of Lodz, 90-549 Lodz, Poland; 2 Department of Rheumatology, Medical University of Lodz, 90-549 Lodz, Poland

**Keywords:** antisynthetase syndrome, diffuse alveolar haemorrhage, interstitial lung disease

## Abstract

**Background:** Diffuse alveolar haemorrhage (DAH) is a rare but potentially life-threatening pulmonary complication of systemic autoimmune diseases. Although interstitial lung disease (ILD) is a hallmark of antisynthetase syndrome (ASyS), DAH has been only exceptionally reported in this setting. **Methods:** We present two patients with ASyS who developed DAH confirmed by bronchoalveolar lavage. **Results:** The first case involved a 52-year-old woman initially diagnosed with rheumatoid arthritis, later reclassified as rheumatoid arthritis–ASyS overlap, who developed DAH in the context of progressive ILD. The second case concerned a 37-year-old man with newly diagnosed ASyS who presented with recurrent DAH early in the disease course. Both patients required high-dose corticosteroids, followed by escalation of immunosuppressive therapy with rituximab or mycophenolate mofetil, resulting in clinical and radiological improvement. These cases illustrate the diagnostic challenges posed by DAH in ASyS, particularly due to overlapping features with infection and ILD exacerbation. They also highlight the importance of early bronchoscopy and timely intensification of immunosuppression. **Conclusions:** Increased awareness of DAH as a rare manifestation of ASyS may facilitate earlier recognition and improve outcomes in patients presenting with acute respiratory deterioration, anaemia, or haemoptysis.

## 1. Introduction

Diffuse alveolar haemorrhage (DAH) is a clinicopathologic syndrome characterised by bleeding into the alveolar spaces originating from the pulmonary microcirculation. The clinical presentation typically includes haemoptysis, anaemia, diffuse alveolar infiltrates, and hypoxaemic respiratory failure. Causes of DAH include ANCA-associated vasculitis, anti-GBM disease, systemic lupus erythematosus, and other connective tissue diseases. Diagnosis relies on clinical, laboratory, radiologic, and pathologic findings [1,2,3]. A rising erythrocyte count in sequential bronchoalveolar lavage (BAL) aliquots and the presence of hemosiderin-laden macrophages are characteristic [4]. Treatment focuses on treating the underlying pathology, with corticosteroids and immunosuppressive agents being the mainstay of therapy [3,4].

Antisynthetase syndrome (ASyS) is a systemic autoimmune disorder within the spectrum of idiopathic inflammatory myopathies (IIMs). It is defined by the presence of antibodies directed against specific aminoacyl-transfer RNA synthetases (ARSs), with anti-Jo-1 (anti-histidyl-tRNA synthetase) being the most common. The estimated prevalence of ASyS is 1/25,000–33,000, with females being affected more frequently than males at a ratio of approximately 2–3:1 [5,6]. The clinical manifestations are heterogeneous and depend on the type of antibodies present. The cardinal symptoms include myositis, arthritis, interstitial lung disease (ILD), Raynaud’s phenomenon, fever, and mechanic’s hands [7,8]. ILD is notable for being the most common extramuscular manifestation of ASyS, with an overall prevalence ranging from 70% to 95% [7,8,9]. In certain antisynthetase antibody subsets, particularly anti-PL-7, anti-PL-12, and anti-EJ, ILD may represent the first or even the sole clinical manifestation. According to the most recent CLASS 2024 criteria, a diagnosis of antisynthetase syndrome can be established in patients presenting exclusively with ILD and antisynthetase antibodies, even in the absence of other clinical features [10].

Patients may present with a chronic course characterised by cough and exertional dyspnoea; however, some patients develop a rapidly progressive form of the disease, which is associated with a poor prognosis. Common HRCT findings in ASyS-associated ILD include bibasilar fibrosis, ground-glass opacities, interlobular reticulation, and traction bronchiectasis. The most common radiographic pattern is non-specific interstitial pneumonia, followed by organising pneumonia and usual interstitial pneumonia [11]. Several sets of diagnostic criteria have been proposed, including Solomon’s criteria, Connor’s criteria, and the CLASS Project [8]. Each relies on the presence of antisynthetase antibodies and cardinal clinical symptoms. Glucocorticoids and immunosuppressive drugs are first-line treatments, but other agents such as cyclophosphamide and rituximab are also used in various clinical scenarios [8,12].

DAH is a rare manifestation of ASyS-associated ILD; however, it may occur in the context of pulmonary capillaritis associated with connective tissue diseases. Although exceptionally uncommon, several case reports have documented DAH in ASyS, including patients with anti-PL-12 and anti-PL-7 antibodies [13,14]. As a potentially life-threatening condition, rapid diagnosis and treatment are essential. This report highlights the clinical importance of ASyS-associated DAH as a rare but significant phenomenon.

## 2. Cases

### 2.1. Case 1

A 52-year-old woman with a history of inflammatory arthritis, initially diagnosed as rheumatoid arthritis (RA) in 2019, was admitted to the pulmonary department because of progressive fatigue, markedly reduced exercise tolerance with oxygen desaturation to 75% on room air, dyspnoea, cough with haemoptysis, and unintentional weight loss of approximately 10 kg over the preceding three months. Her medical history also included hypertension, hypercholesterolemia, gastroesophageal reflux disease, and a 35-pack-year smoking history. At the time of admission, she was receiving leflunomide 20 mg/day. Previously, she had been treated with subcutaneous methotrexate (2019–November 2024); leflunomide (20 mg/day, March 2019–April 2020), which was discontinued due to gastrointestinal adverse effects and lack of efficacy; hydroxychloroquine (April 2022–January 2023); adalimumab (March 2020–April 2023), discontinued because of secondary loss of response; and tocilizumab (July 2023–November 2024). Glucocorticosteroids had been used intermittently during disease flares. Leflunomide was reintroduced in August 2024 due to worsening joint pain.

On admission, the patient was in moderately impaired general condition. Peripheral oxygen saturation was 70% on room air. Lung auscultation revealed bilateral basal crackles, more pronounced on the left side.

Laboratory investigations showed elevated inflammatory markers (erythrocyte sedimentation rate 53 mm/h, C-reactive protein 9.4 mg/dL), hypoalbuminemia, and positive anti-HCV antibodies with undetectable HCV RNA. After consultation with an infectious disease specialist, these findings were interpreted as indicative of a past, resolved HCV infection. Immunological testing (ENA3 panel) revealed high-titre anti–Ro-52 antibodies. Antineutrophil cytoplasmic antibodies (p-ANCA and c-ANCA) were negative. Ultrasonography of the parotid glands showed no features suggestive of Sjögren’s disease, and the patient denied sicca symptoms.

**Figure 1:**
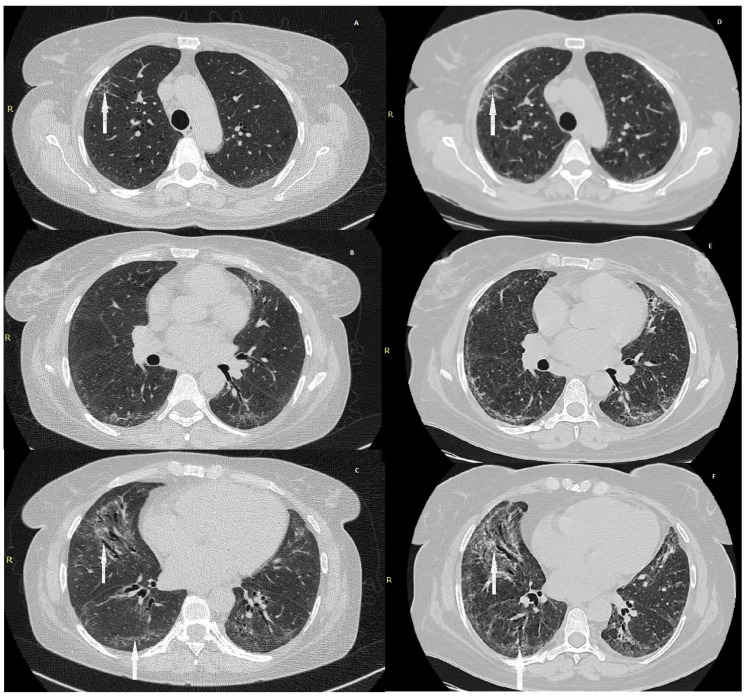
(**A**–**C**): High-resolution computed tomography (HRCT) at initial presentation. Subpleural reticulation, interlobular septal thickening, and early traction bronchiectasis are visible, consistent with fibrotic interstitial lung disease. Arrows indicate areas of subpleural reticulation, septal thickening, and early traction bronchiectasis. (**D**–**F**): Follow-up HRCT showing progression of fibrotic remodelling, with increased reticulation and architectural distortion. Arrows indicate progression of traction bronchiectasis and fibrotic thickening.

Pulmonary function tests demonstrated a restrictive ventilatory defect, with a total lung capacity (TLC) of 1.65 L (71% of predicted) and a severely reduced diffusing capacity for carbon monoxide (DLCO 30% of predicted). High-resolution computed tomography (HRCT) of the chest revealed interlobular septal thickening, bilateral ground-glass opacities, and bronchiectasis (Figure 1A–C).

During hospitalisation, flexible bronchoscopy with bronchoalveolar lavage (BAL) was performed. Microbiological cultures, including acid-fast bacilli staining and PCR testing for *Mycobacterium tuberculosis*, were negative. BAL cytology showed 99% macrophages, the majority containing hemosiderin, and 1% granulocytes, with no atypical cells identified.

Based on the clinical presentation, radiological findings, and BAL results, a diagnosis of diffuse alveolar haemorrhage was established.

The patient was treated with intravenous methylprednisolone pulse therapy (1 g daily for three consecutive days), followed by oral prednisone 60 mg/day (approximately 1 mg/kg). Empirical antibiotic therapy with clarithromycin and trimethoprim–sulfamethoxazole was initiated. Following treatment, the patient’s clinical condition improved significantly, with resolution of respiratory failure. Resting oxygen saturation increased to 97% on room air, with exercise-induced desaturation to 90%.

After one month of glucocorticoid therapy, the patient was readmitted for clinical and radiological reassessment. She reported marked improvement and denied dyspnoea, cough, or fever. Oxygen saturation was 93% while breathing room air. Follow-up HRCT demonstrated persistent interlobular septal thickening with partial regression of bilateral ground-glass opacities and bronchiectasis. Fibrotic changes involved approximately 40% of total lung parenchyma (Figure 1D–F).

Nailfold capillaroscopy revealed a normal pattern. Repeat pulmonary function testing showed forced vital capacity (FVC) of 84% predicted and DLCO (single-breath method) of 45% predicted. Assessment of inflammatory arthritis activity demonstrated remission (DAS28-ESR 2.38). At the time of DAH onset, rheumatoid arthritis activity was low (DAS28-ESR 2.38), with no clinical synovitis and no radiographic erosions. Rheumatoid factor was negative, and anti-CCP antibodies were absent. The overall clinical picture was, therefore, not fully compatible with active RA, which prompted reconsideration of the underlying diagnosis once ILD and mechanic’s hands emerged.

Given the clinical picture of diffuse alveolar haemorrhage associated with interstitial lung disease, immunosuppressive therapy was escalated to rituximab.

During a subsequent evaluation for antifibrotic therapy in the pulmonology department, the patient underwent extended rheumatological and immunological reassessment. Myositis3 panel revealed strongly positive anti-Jo1, moderately positive anti-HMGCR, and weakly positive anti-SAE1 and anti-PL7 antibodies. Based on the presence of interstitial lung disease, inflammatory arthritis, mechanic’s hands, and serological profile, the initial diagnosis of connective tissue disease–associated interstitial lung disease was revised. Following rheumatological consultation, the patient was ultimately diagnosed with an overlap syndrome of rheumatoid arthritis and antisynthetase syndrome, which was considered the underlying cause of her clinical manifestations. In retrospect, the episode of diffuse alveolar haemorrhage was interpreted as pulmonary involvement in the course of antisynthetase syndrome rather than rheumatoid arthritis alone.

Currently, the patient remains in a relatively stable condition on moderate-dose steroid therapy, rituximab, nintedanib, and home oxygen therapy, awaiting lung transplantation. She has been formally evaluated and accepted for lung transplantation at a transplant centre and is now actively listed and awaiting the procedure.

### 2.2. Case 2

A 37-year-old man with no prior history of chronic diseases was admitted to the pulmonary department for evaluation of abnormal findings on a chest X-ray performed two weeks earlier. The X-ray revealed bilateral reticulonodular opacities. Imaging was obtained due to haemoptysis, exertional dyspnoea, fatigue, polyarticular arthralgia without joint swelling, chronic cough exacerbated by sudden temperature changes, unintentional weight loss of approximately 3 kg, and low-grade fever accompanied by nocturnal diaphoresis over the preceding two months.

On admission, the patient was in good general condition, without dyspnoea at rest, peripheral oedema, pleuritic chest pain, epistaxis, or signs of gastrointestinal or urinary tract bleeding. Laboratory tests revealed normocytic anaemia, with a haemoglobin level of 8.7 g/dL, and elevated inflammatory markers (CRP 90 mg/L), along with hypoxemia (pO_2_ 50.4 mmHg; oxygen saturation 85.7%). A chest computed tomography (CT) scan demonstrated bilateral peripheral reticulation with areas of honeycombing and ground-glass opacities, predominantly in the basal regions of both lungs.

Bronchofiberoscopy (BF) with bronchoalveolar lavage (BAL) was performed, yielding increasingly bloody sequential aliquots, confirming the diagnosis of DAH. Bronchoaspirate samples were collected for cytological and microbiological analysis. Differential cell count revealed 47% lymphocytes, 3% macrophages, 50% neutrophils, and numerous erythrocytes. Cultures grew *Streptococcus* spp. group C, and *Pneumocystis jirovecii* genetic material was detected. Given the detection of *Pneumocystis jirovecii* DNA, prophylactic-therapeutic trimethoprim–sulfamethoxazole was initiated, although the subsequent clinical course and repeat BAL suggested colonisation rather than active infection. Antibiotic therapy with ceftriaxone and levofloxacin was initiated, and antihaemorrhagic drugs as well as intravenous prednisolone were administered. This resulted in clinical improvement, including resolution of haemoptysis and subjective symptoms as well as a reduction in inflammatory markers.

**Figure 2:**
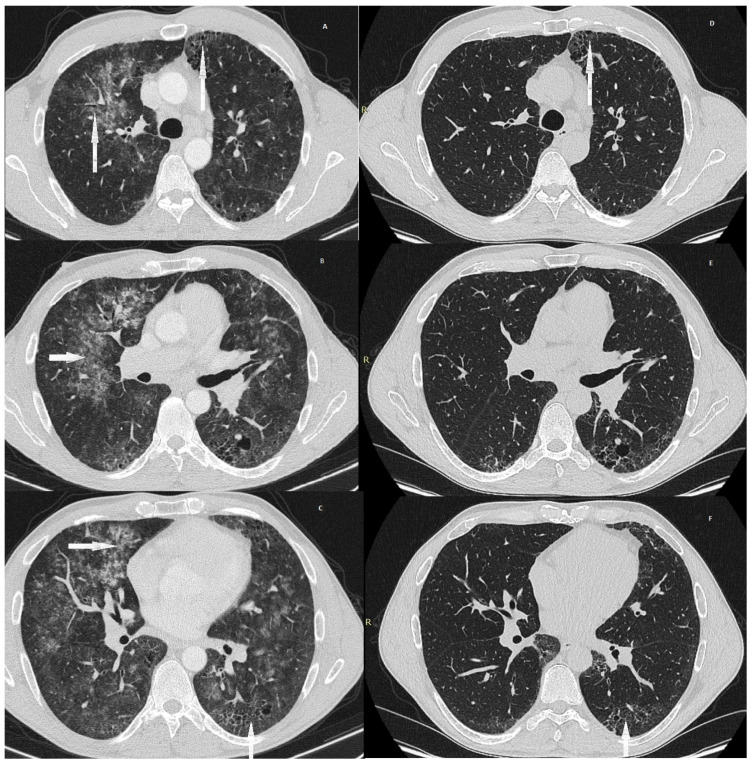
(**A**–**C**): High-resolution computed tomography (HRCT) during a recurrent episode of diffuse alveolar haemorrhage (DAH). Diffuse bilateral ground-glass opacities are present, superimposed on pre-existing reticulation. Arrows highlight areas of fresh ground-glass opacities consistent with acute alveolar haemorrhage, underlying reticulation reflecting chronic interstitial lung disease and small foci of honeycombing where visible. (**D**–**F**): Follow-up HRCT demonstrating complete resolution of acute alveolar infiltrates. Persistent reticulation and traction bronchiectasis remain, consistent with fibrotic remodelling. Arrows indicate residual reticulation and traction bronchiectasis.

Two weeks after admission, the patient’s condition deteriorated, with worsening cough and the onset of nocturnal fever. A follow-up chest CT scan showed progression of radiological abnormalities, with extensive bilateral reticulation and ground-glass opacities consistent with recurrent DAH (Figure 2A–C). Areas of emphysema and small foci of honeycombing were also noted, along with multiple mildly enlarged mediastinal and hilar lymph nodes. A single pulse dose of methylprednisolone 500 mg was administered.

The patient was subsequently transferred to a regional pulmonology centre for further diagnostic and therapeutic management. On admission, laboratory evaluation revealed markedly elevated inflammatory markers (CRP 204 mg/L, ESR 77 mm/h), persistent anaemia (haemoglobin 9.0 g/dL), and hypoxemia (pO_2_ 51.7 mmHg; oxygen saturation 84.7%). Intravenous corticosteroid therapy was continued, and mycophenolate mofetil was initiated. Due to ongoing anaemia, two units of packed red blood cells were transfused.

Repeat BF with BAL was performed. The bronchial mucosa was erythematous, consistent with active inflammation. BAL fluid was bloody and contained numerous hemosiderin-laden macrophages. Total cell count was 20 × 10^6^, with 21% neutrophils, 66% macrophages, 10% lymphocytes, and 2% eosinophils. BAL cultures grew *Streptococcus viridans* and *Candida albicans*, while PCR testing for *Pneumocystis jirovecii* was negative. Empirical antibiotic therapy with cotrimoxazole and piperacillin/tazobactam was initiated.

Immunological testing revealed antinuclear antibodies (ANA) at a titre of 1:2560 with a speckled and mitochondrial fluorescence pattern. ENA 3 Profile and Myositis 3 Profile testing demonstrated strongly positive anti-Jo-1 and anti-AMA-M2 antibodies and positive anti-Ku and anti-HMGCR antibodies, and moreover, weakly positive anti-CCP antibodies were detected. Anti-GBM and c-ANCA and c-ANCA antibodies were absent. Unlike Patient 1, Patient 2 tested negative for anti-Ro52 antibodies, which have been associated with more severe ILD in antisynthetase syndrome.

**Figure 3:**
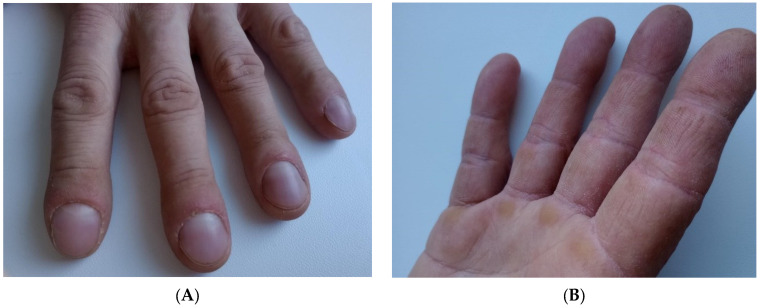
(**A**,**B**) Hands of the patient demonstrating classic mechanic’s hands associated with antisynthetase syndrome: marked hyperkeratosis, fissuring, and rough scaling along the radial aspects of the fingers and the ulnar borders of the thumbs. The skin shows a coarse texture with irregular cracking and accentuated dermatoglyphics, consistent with chronic hyperkeratotic changes characteristic of this autoimmune phenotype.

Rheumatological consultation revealed subtle bilateral Gottron’s signs over the metacarpophalangeal and elbow joints as well as hyperkeratosis of the lateral and palmar parts of the fingers consistent with mechanic’s hands (Figure 3A,B). Based on the clinical presentation and serological findings, a diagnosis of antisynthetase syndrome (ASyS) was established. The patient fulfilled both the 2024 CLASS criteria and the Solomon’s criteria for antisynthetase syndrome.

Electroneurography (ENG) and electromyography (EMG) were performed and demonstrated normal nerve conduction parameters, with no evidence of myogenic or neurogenic muscle involvement. Treatment resulted in improvement of laboratory parameters, including an increase in haemoglobin to 11.4 g/dL and a decrease in CRP to 34.5 mg/L, along with gradual clinical improvement. Intravenous corticosteroids were transitioned to oral prednisone, and mycophenolate mofetil was continued. The patient was discharged in good condition on oral prednisone 35 mg daily with a planned taper and mycophenolate mofetil 2 g daily.

At the outpatient follow-up five days after discharge, the patient was asymptomatic, with oxygen saturation of 96% on room air and bibasilar crackles on auscultation. Pulmonary function testing revealed no airway obstruction, with an FEV_1_ of 4.01 L (91% predicted) and an FVC of 4.73 L (86% predicted). However, single-breath diffusing capacity for carbon monoxide (TLCO-SB K) was reduced to 5.07 mmol/min/kPa (52% predicted).

A subsequent follow-up hospitalisation one month later demonstrated further clinical improvement, with increased exercise tolerance and resolution of cough. The patient denied dyspnoea, chest pain, haemoptysis, weight loss, or systemic symptoms. Physical examination revealed normal bilateral vesicular breath sounds with persistent basal crackles. Oxygen saturation was 98% on room air.

Chest CT showed regression of ground-glass opacities in the upper lobes, with persistent peripheral reticulation, ground-glass opacities, and traction bronchiectasis in the lower lobes (Figure 2D–F). Pulmonary function testing showed no airway obstruction (FEV_1_ 3.83 L [86% predicted], FVC 4.88 L [89% predicted], FEV_1_/FVC 78.5%). Body plethysmography revealed reduced inspiratory capacity (2.42 L, 61% predicted), reduced vital capacity (4.49 L, 78% predicted), and total lung capacity near the lower limit of normal (6.10 L, 83% predicted). Lung diffusion testing demonstrated increased residual volume (2.75 L, 170% predicted), elevated RV/TLC ratio (36.5%, 167% predicted), and persistently reduced TLCO-SB K (6.36 mmol/min/kPa, 62% predicted), consistent with restrictive ventilatory impairment due to ASyS-associated interstitial lung disease.

Given the sustained clinical improvement, partial radiological regression, and improvement in functional parameters, further tapering of prednisone was recommended while continuing mycophenolate mofetil. The patient remains in stable condition under combined pulmonological and rheumatological care. Arthralgia and mechanic’s hands resolved. The latest nailfold capillaroscopy demonstrated the presence of bushy capillaries, indicating neoangiogenesis, frequently observed in IIM patients.

## 3. Discussion

Diffuse alveolar haemorrhage (DAH) is a rare but potentially life-threatening pulmonary manifestation of systemic autoimmune diseases. It is most commonly observed in association with small-vessel vasculitides, systemic lupus erythematosus, and anti-glomerular basement membrane disease [4]. In contrast, DAH is rarely reported in idiopathic inflammatory myopathies and remains an exceptionally uncommon complication of antisynthetase syndrome (ASyS) [4,15].

Antisynthetase syndrome is a heterogeneous autoimmune condition defined by the presence of antibodies against aminoacyl-tRNA synthetases and characterised clinically by interstitial lung disease, inflammatory arthritis, myositis, Raynaud’s phenomenon, fever, and mechanic’s hands [7,8]. Among these features, ILD represents the principal determinant of prognosis and mortality. While chronic or subacute ILD is well recognised in ASyS, acute pulmonary complications such as DAH are rarely described and may be underdiagnosed due to overlapping clinical and radiological features.

In the present case series, we describe two patients with ASyS-associated lung disease who developed DAH confirmed by bronchoalveolar lavage. Both patients presented with haemoptysis, hypoxemic respiratory failure, and diffuse ground-glass opacities on HRCT, initially raising suspicion of infection or progression of ILD. In both cases, extensive microbiological and immunological evaluation excluded alternative causes, and BAL findings demonstrating hemosiderin-laden macrophages or increasingly bloody aliquots were crucial for establishing the diagnosis of DAH (Table 1).

The first patient represents a diagnostically challenging overlap syndrome. Initially diagnosed with rheumatoid arthritis, she later developed ILD, mechanic’s hands, and a high-titre of anti-Jo1 and anti–Ro-52 antibodies. Anti–Ro-52 has been increasingly recognised as a marker associated with severe and progressive ILD in myositis-spectrum disorders, even in the absence of classical antisynthetase antibodies [16,17]. Retrospective reassessment led to the diagnosis of RA–ASyS overlap syndrome, with DAH interpreted as a manifestation of pulmonary capillaritis related to ASyS rather than RA alone. This case highlights the importance of reconsidering the underlying systemic diagnosis in patients with atypical pulmonary complications and evolving autoimmune features.

The second patient fulfilled established classification criteria for antisynthetase syndrome, with strongly positive anti-Jo-1 antibodies, mechanic’s hands, subtle Gottron’s signs, and ILD. DAH occurred early in the disease course and was recurrent despite initial antimicrobial and corticosteroid therapy. The presence of concomitant infection complicated the diagnostic process; however, persistence and recurrence of haemorrhage together with immunological findings supported an immune-mediated mechanism. Escalation of immunosuppression with pulse corticosteroids and mycophenolate mofetil resulted in sustained clinical, laboratory, and radiological improvement.

Although Patient 2 had weakly positive anti-CCP antibodies, he did not exhibit clinical synovitis, erosive disease, or other features required to meet the European Alliance of Associations for Rheumatology (EULAR)/American College of Rheumatology (ACR) classification criteria for rheumatoid arthritis. The serologic finding was, therefore, interpreted as non-specific and insufficient to support a diagnosis of RA.

The presence of subtle Gottron’s signs and mechanic’s hands raised the question of amyopathic dermatomyositis (ADM). However, the absence of hallmark cutaneous features, such as heliotrope rash or shawl sign, together with strongly positive anti-Jo-1 antibodies supported a diagnosis of antisynthetase syndrome rather than ADM.

Antisynthetase antibodies, which target aminoacyl-tRNA synthetases, are considered an immunological hallmark of antisynthetase syndrome (ASyS). The most prevalent are anti-Jo1 antibodies, directed against histidyl-tRNA synthetase, which are detected in approximately 20–30% of patients with idiopathic inflammatory myopathies (IIM) [18], including both of our presented cases. The remaining antisynthetase antibodies include anti-PL-7 (threonyl-), anti-PL-12 (alanyl-), anti-EJ (glycyl-), anti-OJ (isoleucyl-), anti-KS (asparaginyl-), anti-Zo (phenylalanyl-), and anti-Ha (tyrosyl-tRNA synthetase) [19]. Notably, only anti-Jo1 antibodies are included in the basic immunoblot ENA3 panel, whereas the less common antisynthetase antibodies are assessed exclusively by dedicated myositis panels. Therefore, if ASyS is suspected based on the clinical presentation, extended antibody testing should be performed.

In addition to antisynthetase antibodies, 30–65% of patients with ASyS are positive for anti-Ro52 antibodies [20,21], which was also observed in our Patient 1. The presence of anti-Ro52 in the ENA3 profile may thus serve as a useful clue prompting further investigation for rare myositis-specific antibodies. Interestingly, anti-Jo1 antibodies have been detected not only in serum but also in bronchoalveolar lavage fluid (BALF), suggesting that cleavage of aminoacyl-tRNA may be initiated within the respiratory tract [22].

The pathophysiological mechanisms underlying DAH in ASyS are not fully understood. Pulmonary capillaritis driven by immune complex deposition, complement activation, and cytokine-mediated endothelial injury is considered the most plausible explanation [4]. The association with high inflammatory activity and severe ILD suggests that DAH may represent an extreme manifestation of pulmonary immune dysregulation in ASyS.

Although antisynthetase syndrome (ASyS) is considered a subtype of idiopathic inflammatory myopathies, muscle involvement is not mandatory for establishing the diagnosis. Muscular manifestations in ASyS range from asymptomatic disease to severely disabling muscle weakness. Other features of ASyS, such as ILD or arthritis, may develop independently, and their severity does not necessarily correlate with the extent of muscle involvement [23]. This observation is consistent with our findings, as both of our patients presented with arthritis, severe pulmonary involvement, mechanic’s hands, and Raynaud’s phenomenon in the absence of clinically apparent muscle symptoms at the time of diagnosis. Notably, the clinical presentation of ASyS may evolve over time, as manifestations from the ASyS spectrum tend to emerge gradually. Frequently, disease onset is mono- or oligosymptomatic (e.g., isolated arthritis in patients with anti-Jo1 antibodies or isolated ILD in patients with anti-PL-7, anti-PL-12, or anti-EJ antibodies), with the complete clinical picture developing only later in the disease course [24]. In the study conducted by the AENEAS collaboration group, isolated arthritis was reported as the initial manifestation in up to 24% of patients with anti-Jo1 antibodies [24]. Similarly, our Patient 1 initially presented with isolated joint inflammation, which led to a diagnosis of rheumatoid arthritis.

The heterogeneity of clinical manifestations and the frequent absence of dominant muscle involvement result in many patients not fulfilling the 2017 EULAR/ACR classification criteria for IIM [24]. In response to this limitation, the CLASS group developed novel classification criteria specifically dedicated to ASyS, presented in Table 2 [10]. Patient 2 was diagnosed with definite ASyS based on the CLASS 2024 criteria. At the time of Patient 1’s diagnosis, these criteria were not yet available, and the diagnosis of ASyS was, therefore, based on clinical judgment; however, retrospective assessment indicates that Patient 1 would also fulfil the CLASS criteria for definite ASyS. From a clinical perspective, these cases emphasise several important points. First, DAH should be considered in ASyS patients presenting with acute respiratory deterioration, haemoptysis, anaemia, or new diffuse ground-glass opacities, even in the presence of infection. Second, bronchoscopy with BAL remains essential for timely diagnosis. Interdisciplinary cooperation between pulmonologists and rheumatologists is important for the proper diagnosis and comprehensive care of patients with systemic connective tissue disease such as ASyS. Finally, prompt initiation or escalation of immunosuppressive therapy is critical and may be lifesaving.

## 4. Conclusions

Diffuse alveolar haemorrhage is a rare but potentially life-threatening pulmonary manifestation of antisynthetase syndrome, including overlap forms of connective tissue diseases. DAH should be considered in patients with ASyS who present with acute respiratory deterioration, haemoptysis, anaemia, or new diffuse ground-glass opacities on imaging, even in the presence of concomitant infection. Early bronchoscopy with bronchoalveolar lavage is essential for accurate diagnosis. Prompt initiation and escalation of immunosuppressive therapy may be lifesaving and can lead to favourable clinical and functional outcomes. Increased awareness of this rare complication may facilitate earlier recognition and improve prognosis in this high-risk patient population.

## Figures and Tables

**Table 1:** Clinical characteristics of patients with antisynthetase syndrome-associated diffuse alveolar haemorrhage.

Feature	Case 1	Case 2
Age/Sex	52/Female	37/Male
Initial diagnosis	Rheumatoid arthritis	None
Final diagnosis	RA–ASyS overlap	Antisynthetase syndrome
Presenting symptoms	Dyspnoea, haemoptysis, weight loss	Haemoptysis, dyspnoea, fever
Mechanic’s hands	Present	Present
Myositis	Absent	Absent
ILD on HRCT	Yes (GGO, fibrosis, bronchiectasis)	Yes (reticulation, honeycombing, GGO)
DAH confirmation	BAL (hemosiderin-laden macrophages)	BAL (bloody aliquots, hemosiderin)
Key autoantibodies	Anti-Jo1, anti–Ro-52	Anti-Jo-1, anti-AMA-M2 (+++)
ANCA/anti-GBM	Negative	Negative
Treatment	Steroids → rituximab	Steroids → MMF
Outcome	Clinical and radiological improvement	Clinical and functional improvement

Abbreviations: ASyS—antisynthetase syndrome; BAL—bronchoalveolar lavage; DAH—diffuse alveolar haemorrhage; GGO—ground-glass opacities; ILD—interstitial lung disease; MMF—mycophenolate mofetil.

**Table 2:** CLASS 2024 criteria for ASyS.

**A.** **Clinical domain**	**Weight**
**Joint involvement**	
Inflammatory arthralgia or arthritis	1.0
**Muscle involvement**	
Subclinical evidence of myositis (muscle enzyme/EMG/MRI)	1.0
Clinical evidence of myositis	1.5
Biopsy-supported myositis	2.0
**Interstitial lung disease**	
UIP or other/unclassifiable pattern	1.5
Predominant NSIP and/or OP pattern	2.5
**B.** **Other clinical features**	**Weight**
**Mechanic’s hand/Hiker’s feet**	1.5
**Raynaud’s phenomenon**	0.5
**Inflammatory rashes** (Gottron’s, heliotrope, V-sign, shawl sign, malar rash)	0.5
**Unexplained fever**	0.5
**C.** **Serological domain**	**Weight**
**Antisynthetase antibodies**	
Non-Jo1 positive by non-IP methods	3.0
Jo1 positive by any methods or non-Jo1 positive by IP	3.5
**ANA with cytoplasmic pattern**	1.5
**Anti-Ro52 or anti-SSA antibodies**	1.0
Count the highest weight from each of the domains: A + B + C **Probable ASyS—total score > 5.0** **Definite ASyS—total score > 5.5**	

Abbrevations: EMG—electromyography, MRI—magnetic resonance imaging, UIP—usual interstitial pneumoniae, NSIP—non-specific interstitial pneumoniae, OP—organising pneumoniae, IP—immunoprecipitation, ANA—antinuclear antibodies.

## Data Availability

No datasets were generated or analysed in this study.

## Abbreviations

The following abbreviations are used in this manuscript:

ASyS	antisynthetase syndrome
DAH	diffuse alveolar haemorrhage
ILD	interstitial lung disease
BAL	bronchoalveolar lavage
HRCT	high-resolution computed tomography
ANA	antinuclear antibodies
MMF	mycophenolate mofetil
RA	rheumatoid arthritis
